# Association between angiotensin II receptor type 1 A1166C polymorphism and chronic kidney disease

**DOI:** 10.18632/oncotarget.24469

**Published:** 2018-02-12

**Authors:** Hsien-Feng Chang, Po-Jen Hsiao, Yu-Juei Hsu, Fu-Huang Lin, Chin Lin, Wen Su, Hsiang-Cheng Chen, Sui-Lung Su

**Affiliations:** ^1^ School of Public Health, National Defense Medical Center, Taiwan, ROC; ^2^ Division of Nephrology, Department of Medicine, Tri-Service General Hospital, National Defense Medical Center, Taiwan, ROC; ^3^ Department of Internal Medicine, Taoyuan Armed Forces General Hospital, Taiwan, ROC; ^4^ Big Data Research Center, Fu-Jen Catholic University, Taiwan, ROC; ^5^ Department of Nursing, Tri-Service General Hospital, Taiwan, ROC; ^6^ Division of Rheumatology/Immunology/Allergy, Department of Internal Medicine, Tri-Service General Hospital, National Defense Medical Center, Taiwan, ROC

**Keywords:** angiotensin II receptor type 1, AGTR1 A1166C, chronic kidney disease, meta-analysis, gene-environment interaction

## Abstract

Studies of the association between angiotensin II receptor type 1 A1166C (AGTR1 A1166C) polymorphism and chronic kidney disease (CKD) risk have yielded conflicting results. We conducted a combined case-control study and meta-analysis to better define this association. The case-control study included 634 end-stage renal disease (ESRD) patients and 739 healthy controls. AGTR1 A1166C genotype was determined using polymerase chain reaction and iPLEX Gold SNP genotyping methods. The meta-analysis included 24 studies found in the PubMed and Cochrane Library databases. Together, the case-control study and meta-analysis included 36 populations (7,918 cases and 6,905 controls). We found no association between the C allele and ESRD (case-control study: OR: 1.02, 95% CI: 0.77–1.37; meta-analysis: OR: 1.07; 95% CI: 0.97–1.18). Co-dominant, dominant, and recessive model results were also not significant. No known environmental factors moderated the effect of AGTR1 A1166C on CKD in our gene-environment interaction analysis. Sensitivity analysis showed an AGTR1 A1166C-CKD association in Indian populations (OR: 1.46, 95% CI: 1.26–1.69), but not in East Asian or Caucasian populations. Additional South Asian studies will be required to confirm the potential role of this polymorphism in CKD.

## INTRODUCTION

Chronic kidney disease (CKD) is highly prevalent worldwide [1–4], and increases patient cardiovascular event and mortality risks [5]. Genetic factors play key roles in CKD pathogenesis. For example, creatinine clearance appears inherited in 46% of cases [6]. Identification and evaluation of novel candidate gene polymorphisms could lead to improved CKD diagnostic accuracies and therapeutic options.

The renin-angiotensin system (RAS) regulates blood pressure and electrolyte balance, and an over-active RAS leads to CKD [7]. RAS functions mainly through its final product, angiotensin II (Ang II), which binds Ang II receptor types 1 (AGTR1) and 2 (AGTR2) [8, 9]. Because AGTR2 is highly expressed in fetal tissues, but only weakly expressed in adult tissues [10, 11], current CKD genetic research focuses on AGTR1. The silent polymorphism, A1166C (rs5186), is located in the human AGTR1 3’-UTR (untranslated region) [12]. This region is recognized by microRNA-155 (miR-155), and patients harboring the A allele exhibit higher miR-155 and AGTR1 levels than those with the C allele [13].

Previous studies found associations between AGTR1 A1166C and coronary artery disease [14] and breast cancer [15], although genome-wide association studies (GWASs) [16–18] found no significant AGTR1-associated signals. However, GWASs are limited because they tend to explain only a small amount of phenotypic variance, making it difficult to mechanistically assign the loss of function of a specific gene to a single polymorphism [19, 20]. These might be attributable to missing heritability, which is hidden in gene–environment interactions [21, 22]. Additionally, the amount of variance attributable to a SNP detected in a GWAS is likely to be less than that attributable to a true causal variant [23]. Although many previous meta-analyses have investigated AGTR1 A1166C polymorphisms in CKD, their sample sizes were small. Additionally, to our knowledge, no studies have considered gene-environment interactions [12, 24]. We performed a case-control study to investigate the association between AGTR1 A1166C and CKD, and a subsequent meta-analysis combining our data with current evidence. Our systematic analysis provides a clearer understanding of the effect of AGTR1 A1166C on CKD.

## RESULTS

### Case-control study

Case and control patient characteristics are shown in Table 1. We included 634 cases with a mean age of 64.5±14.9 years (296 men and 338 women) and 739 controls with a mean age of 72.7±7.2 years (298 men and 441 women). Mean age was higher among controls relative to cases (p=0.001), and sex distribution differed between the groups (p=0.015). The control group had a higher average BMI than the case group (p=0.001).

**Table 1:** ESRD and control subject characteristics

	Case (N=634)	Control (N=739)	p-value
**Age (years)**	64.5±14.9	72.7±7.2	<0.001
**Sex (% male)**	296 (46.7%)	298 (40.2%)	0.015
**BMI (kg/m^2^)**	22.4±4.0	24.1±3.2	<0.001
**Hypertension**	332 (57.8%)	303 (40.8%)	<0.001
**DM**	213 (54.2%)	91 (12.3%)	<0.001
**TC (mg/dL)**	166.0±36.1	191.0±32.5	<0.001
**TG (mg/dL)**	158.5±109.7	116.1±60.2	<0.001
**Creatinine (mg/dL)**	9.6±2.5	0.8±0.2	<0.001
**eGFR (ml/min/1.73m^2^)**	5.5±1.9	90.5±15.7	<0.001
**Smoke**	122 (21.1%)	76 (10.3%)	<0.001

Abbreviations: BMI: body-mass index; DM: diabetes mellitus; TC: total cholesterol; TG: triglycerides; eGFR: estimated glomerular filtration rate.

A control sample cannot be genotyped, so the genotyping call rate in this study is 99.9%. Now there are only 634 cases and 738 controls in further analysis. We used four types of genetic models to test the association between AGTR1 A1166C and ESRD; the result is shown in Table 2. AGTR1 A1166C C allele frequencies were 6.4% and 6.2% in cases and controls, respectively. The C allele-CKD association was nonsignificant (OR: 1.02; 95% CI: 0.77–1.37). Results from the co-dominant, dominant, and recessive models were also nonsignificant. We further analyzed the association between AGTR1 A1166C and ESRD after adjusting age, sex, BMI, hypertension, DM, and smoking, because these factors differed between controls and cases. However, multivariate analysis results were nonsignificant.

**Table 2:** AGR1 A1166C genotype frequencies in cases and controls

	Case	Control	Crude-OR (95% CI)	p-value	Adj-OR (95% CI)	p-value
**Allele**						
**A**	1187 (93.6%)	1384 (93.8%)	1	0.876	1	0.998
**C**	81 (6.4%)	92 (6.2%)	1.02 (0.77–1.37)		1.00 (0.62–1.60)	
**Co-dominant**						
**AA**	560 (88.3%)	656 (88.9%)	1	0.816	1	0.429
**AC**	67 (10.6%)	72 (9.8%)	1.09 (0.77–1.55)		1.25 (0.70–2.22)	
**CC**	7 (1.1%)	10 (1.4%)	0.82 (0.31–2.17)		0.43 (0.09–2.17)	
**Dominant**						
**AA**	560 (88.3%)	656 (88.9%)	1	0.676	1.00	0.293
**AC+CC**	74 (11.7%)	82 (11.1%)	0.81 (0.31–2.15)		0.42 (0.08–2.11)	
**Recessive**						
**AA+AC**	627 (98.9%)	728 (98.6%)	1	0.744	1.00	0.703
**CC**	7 (1.1%)	10 (1.4%)	1.06 (0.76–1.48)		1.11 (0.64–1.93)	

OR: odds ratio; CI: confidence interval; Adj-OR: odds ratio after adjusting for age, sex, BMI, hypertension, DM, and smoking habits.

### Meta-analysis

**Figure 1:**
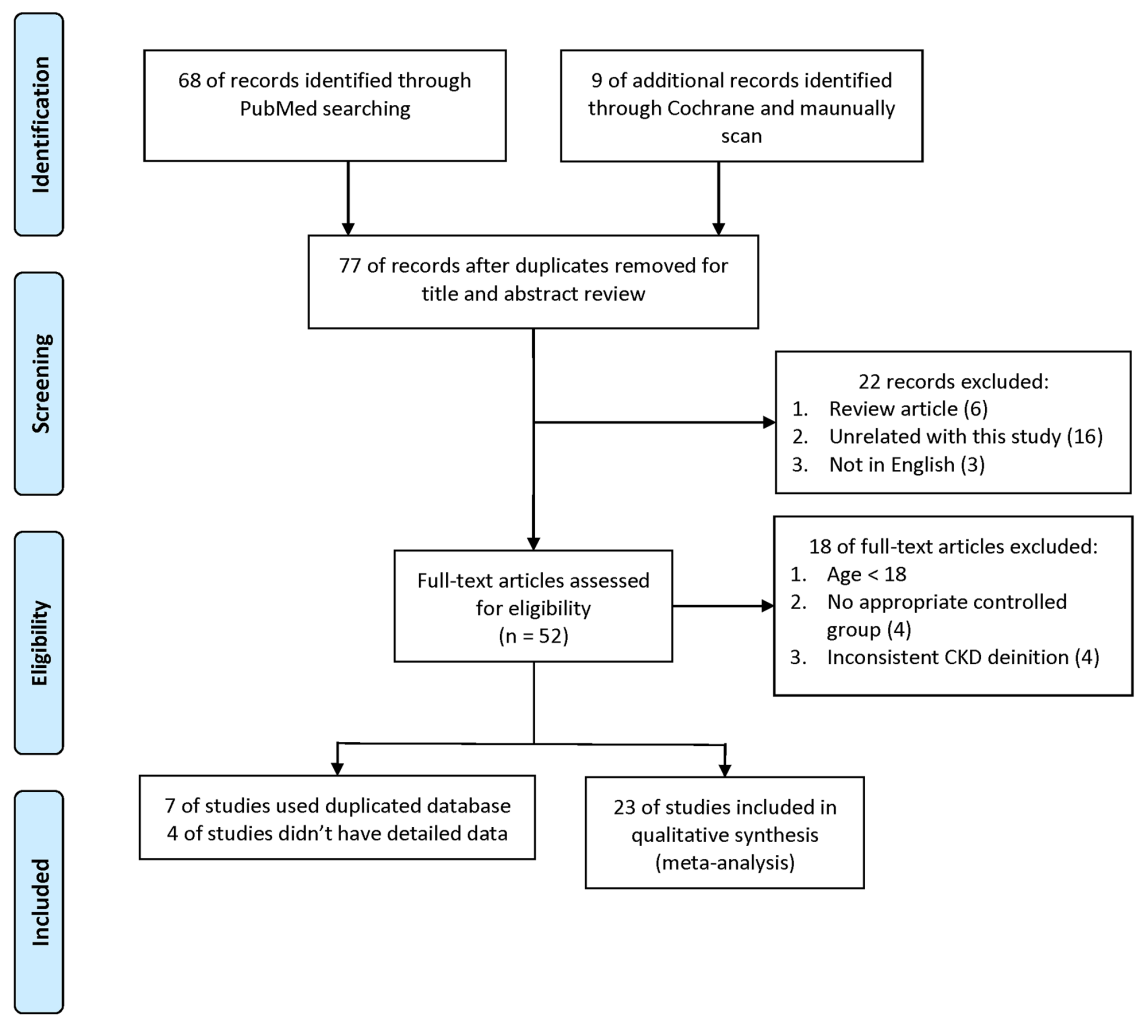
Meta-analysis study identification process

The study identification process is shown in Figure 1. For AGTR1 A1166C and CKD, our search returned 77 records from PubMed, the Cochrane Library, and manual scans. We excluded 25 papers after a preliminary review of the titles and abstracts. An additional 18 papers were excluded after assessing the full-text articles, leaving 34 articles that matched our criteria. Of these, seven used duplicate databases and four lacked detailed data. Of the 23 studies that were ultimately included [25–47], Thomas, *et al.* [42], Kim, *et al.* [33], Mollsten, *et al.* [31], and Shah, *et al.* [29] reported results in the context of stratification. Our meta-analysis of AGTR1 A1166C and CKD therefore comprised 35 populations, including our case-control study (Supplementary Table 3).

**Figure 2:**
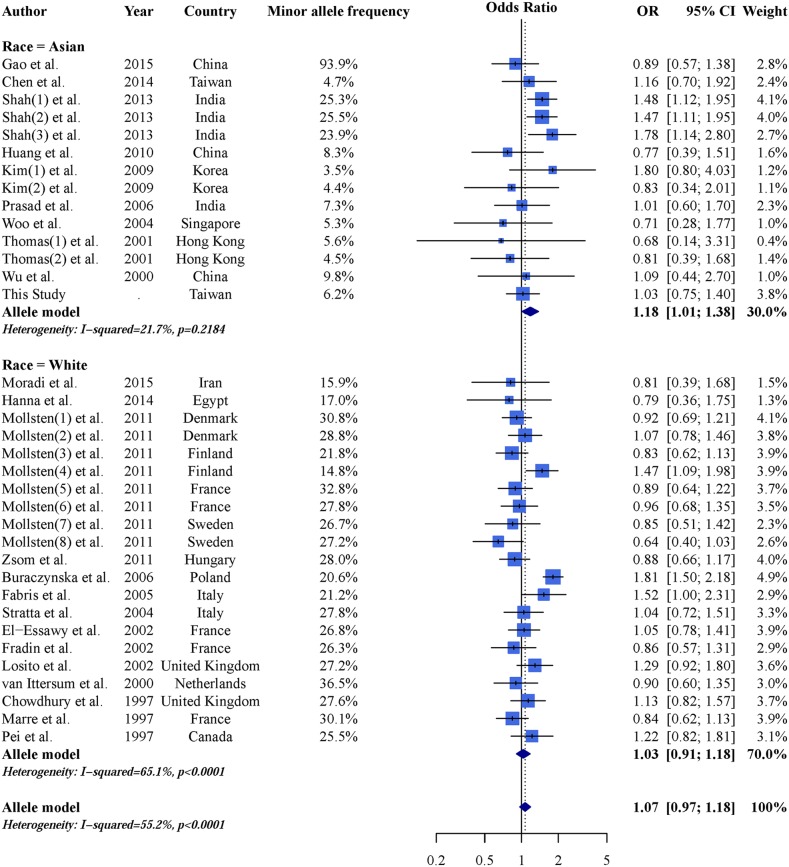
Allele model AGTR1 A1166C and CKD forest plot

**Figure 3:**
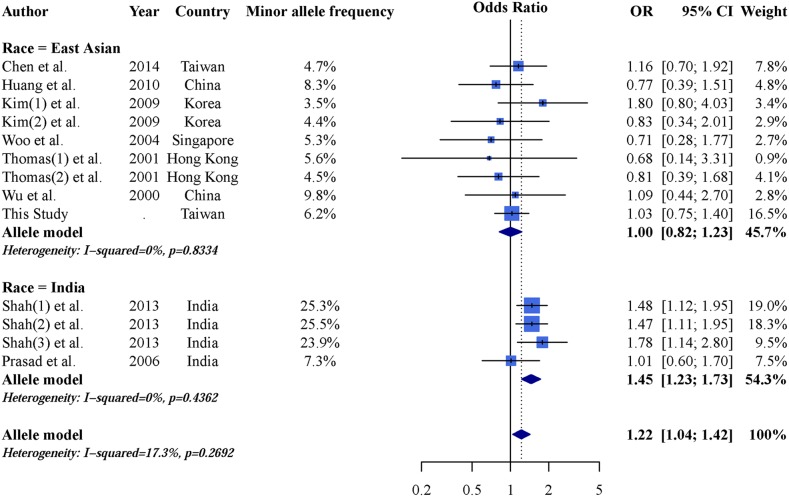
Allele model forest plot of AGTR1 A1166C and CKD in east Asian and Indian populations

An AGTR1 A1166C and CKD forest plot was calculated using the allele model (Figure 2). The overall pooled result showed no significant association between AGTR1 A1166C and CKD (OR: 1.07; 95% CI: 0.97–1.18), and a subgroup analysis found no significant results in Caucasian populations (OR: 1.03; 95% CI: 0.91–1.18). However, the C allele is a CKD risk factor in Asian populations (OR: 1.18; 95% CI: 1.01–1.38). These results demonstrate heterogeneity and indicate the need for further analysis. Dominant and recessive model results were similar to those of the allele model (Table 3). Careful examination identified three studies [27, 29, 34] that were not suitable for inclusion in the Asian subgroup. Gao, *et al.* [27] reported a 93.9% minor allele frequency in a Chinese population, which differed greatly from other Chinese studies and the 1,000 Genomes Database [48]. Additionally, Asian populations can be subdivided into East and South Asians, whose allele frequencies may differ [48]. Thus, we included two studies [29, 34] from India within an independent subgroup. Result were not significant in the East Asian subgroup (OR: 1.00; 95% CI: 0.82–1.23), but were in the Indian subgroup (OR: 1.45; 95% CI: 1.23–1.73) (Figure 3). After further stratification, heterogeneities in these two subgroup disappeared. Dominant and recessive model results were similar to those of the allele model (Table 4).

**Table 3:** AGTR1 A1166C and CKD odds ratios using allele, dominant, and recessive model assumptions

Model	Total				Asian				White			
	OR	95% CI	I^2^	Egger's test	OR	95% CI	I^2^	Egger's test	OR	95% CI	I^2^	Egger's test
**Overall analysis**												
Allele (C vs. A)	1.07	0.97–1.18	55.2%	0.367	1.18	1.01–1.38	21.7%	0.205	1.03	0.91–1.18	65.1%	0.033
Dominant (CC + AC vs AA)	1.06	0.94–1.20	51.3%	0.040	1.17	1.00–1.36	0.0%	0.060	1.02	0.87–1.20	64.8%	0.024
Recessive (CC vs AA +AC)	1.26	0.96–1.65	60.0%	0.118	2.31	0.96–5.54	77.7%	0.095	1.10	0.84–1.44	49.2%	0.657

Abbreviations: I^2^: index of study heterogeneity; Egger's test: p-value of Egger's regression for asymmetry assessment.

**Table 4:** AGTR1 A1166C and CKD odds ratios in east Asian and Indian populations using allele, dominant, and recessive model assumptions

Model	East Asian^a^				Indian			
	OR	95% CI	I^2^	Egger's test	OR	95% CI	I^2^	Egger's test
**Sensitivity analysis**								
Allele (C vs. A)	1.00	0.82; 1.23	0%	0.504	1.45	1.23–1.73	0%	0.603
Dominant (CC + AC vs AA)	1.01	0.81; 1.25	0%	0.387	1.36	1.09–1.68	0%	0.792
Recessive (CC vs AA +AC)	0.81	0.31; 2.15	0%	NA^b^	4.95	2.66–9.20	0%	0.239

^a^: Sensitivity analysis excluded three studies as described in Results.

^b^: Analysis included only one population.

We used meta-regression to detect potential gene-environment interactions that could explain the high heterogeneity among these studies (Table 5). None of the investigative factors moderated the effect of AGTR1 A1166C on CKD. We also excluded the three studies deemed unsuitable for inclusion in the Asian subgroup [27, 29, 34] to avoiding confounding effects. Table 6 shows the meta-regression sensitivity analysis results. DM prevalence is the only significant moderator after Bonferroni correction (OR: 0.58; 95% CI: 0.43–0.78; p<0.001), but Egger's regression analysis shows asymmetry in this result (p=0.008). Because a previous study considered asymmetry to imply some bias [49], we concluded that our meta-regression results were not robust. In summary, no investigative factors moderated the effects of AGTR1 A1166C on CKD.

**Table 5:** Moderator effects of the allele model (C vs. A) on AGTR1 A1166C and CKD

	n	τ^2^	Adjust τ^2^	OR	95% CI	p-value^$^	Egger's test p-value
Race	35	0.0452	0.0460	0.90	0.72–1.12	0.345	0.011
Study design	35	0.0452	0.0423	1.09	0.90–1.34	0.382	0.039
Quality score (per 1 score)	35	0.0452	0.0425	1.08	0.97–1.19	0.152	0.063
Kidney function of case	35	0.0452	0.0341	1.21	0.95–1.54	0.129	0.108
Gender (per 100%)	33	0.0480	0.0502	1.00	0.70–1.42	0.977	0.087
Age (per 10 year)	34	0.0473	0.0436	1.09	0.98–1.20	0.104	0.093
BMI (per 5 kg/m^2^)	11	0.0393	0.0488	0.95	0.65–1.38	0.790	0.263
Hypertension (per 100%)	29	0.0552	0.0487	1.63	0.80–3.32	0.178	0.064
DM (per 100%)	25	0.0603	0.0352	0.69	0.48–1.00	0.051	0.017
Smoke (per 100%)	11	0.0102	0.0123	1.58	0.34–7.41	0.562	0.355

Dependent variable: log odds ratio of AGTR1 A1166C and CKD using allele model.

Abbreviations: n: number of studies; OR: odds ratio for moderator effects; CI: confidence interval.

Race: Asian is reference; study design: cross-sectional study is reference; kidney function of case: not only ESRD patients as reference; gender: proportion of males; age: mean age; BMI: body mass index; hypertension: hypertension prevalence; DM: diabetes mellitus prevalence; Smoke: smoking prevalence.

^$^: P<0.05/10 is considered significant due to Bonferroni correction.

**Table 6:** Meta-regression sensitivity analysis

	n	τ^2^	Adjust τ^2^	OR	95% CI	p-value^$^	Egger's test p-value
Race	30	0.0429	0.0448	1.05	0.78–1.41	0.747	0.090
Study design	30	0.0429	0.0264	1.24	1.02–1.50	0.031	0.027
Quality score (per 1 score)	30	0.0429	0.0462	1.01	0.89–1.16	0.848	0.109
Kidney function of case	30	0.0429	0.0205	1.30	1.05–1.62	0.017	0.198
Gender (per 100%)	28	0.0462	0.0490	1.00	0.70–1.43	0.999	0.142
Age (per 10 years)	29	0.0452	0.0470	1.04	0.93–1.17	0.458	0.134
BMI (per 5 kg/m^2^)	8	0.0110	0.0210	0.93	0.67–1.30	0.687	0.520
Hypertension (per 100%)	25	0.0545	0.0366	1.96	0.98–3.92	0.057	0.117
DM (per 100%)	20	0.0736	0.0121	0.58	0.43–0.78	< 0.001	0.008
Smoke (per 100%)	11	0.0102	0.0123	1.58	0.34–7.41	0.562	0.355

Dependent variable: log odds ratio of AGTR1 A1166C and CKD using allele model.

Abbreviations: n: number of studies; OR: odds ratio for moderator effects; CI: confidence interval.

Race: Asian is reference; study design: cross-sectional study is reference; kidney function of case: not only ESRD patients as reference; gender: proportion of males; age: mean age; BMI: body mass index; hypertension: hypertension prevalence; DM: diabetes mellitus prevalence; smoke: smoking prevalence.

^$^: P<0.05/10 is considered significant due to Bonferroni correction.

## DISCUSSION

Both our case-control study and meta-analysis revealed no association between AGTR1 A1166C and ESRD. Subgroup analyses identified an association between AGTR1 A1166C and CKD among Asian populations, but this was not significant in East Asians. Although patients with the AGTR1 A1166C A allele exhibit higher miR-155 and AGTR1 levels than those with the C allele [13], and this polymorphism is associated with coronary artery disease [14] and breast cancer [15], current evidence does not support an association between AGTR1 A1166C and CKD in East Asians.

The AGTR1 A1166C-CKD association in the Asian patient subgroup might be attributable to the Indian population, based on sensitivity analysis. Although previous meta-analyses included Indian studies within the Asian subgroup [12, 24], the 1,000 Genomes Project separated South and East Asians into different racial categories [48]. Heterogeneity in our study disappeared after this same classification. Additionally, the association between RAS polymorphism and CKD is complex, and includes gene-gene [50] and gene-environment [51] interactions. Thus, a small racial difference might increase heterogeneity. Moreover, the observed high level of heterogeneity in Caucasians implies that some factor may moderate the effects of AGTR1 A1166C on CKD, but our attempts to explain this heterogeneity using all known factors did not yield significant findings. Future research should focus on the effects of gene-gene interactions on CKD, and additional studies conducted in South Asia will better illustrate the role of AGTR1 A1166C in CKD.

This study had several limitations. First, we relied on tabular data for the meta-analysis, rather than individual patient data. However, a previous study revealed that the inclusion of summary data could increase the sample size and improve confidence [52]. Additionally, our case-control study and meta-analysis yielded similar results. Second, gene-environment interaction analyses are limited by the availability of published data. For example, the included studies each only reported a few factors. Third, gene-gene interactions are difficult to analyze in the context of a meta-analysis. Although our previously-developed method addresses this problem [53], published papers seldom simultaneously report the same polymorphisms. We will conduct a multi-loci meta-analysis in the event that sufficient papers are published.

In conclusion, our meta-analysis found that the AGTR1 A1166C-CKD association was only significant in the Indian subgroup, and more South Asian studies are needed to confirm this finding. The AGTR1 A1166C C allele is likely not a CKD risk factor in East Asians and Caucasians. The observed high level of heterogeneity among studies suggests a complex underlying mechanism that appears unrelated to patient sex, age, BMI, DM, hypertension, or smoking habits. Because this unexplainable heterogeneity might be related to gene-gene interactions, an AGTR1 A1166C-CKD epistasis analysis should be conducted. Some RAS polymorphisms, such as angiotensin converting enzyme I/D and angiotensinogen M235T, may moderate the association between AGTR1 A1166C and CKD. Finally, potential gene-environment interactions may influence this association, and additional environmental factors should be investigated.

## MATERIALS AND METHODS

### Case-control study population size

Based on the following thresholds, the minimum required study sample size was 1047 subjects: two-sided test with a power (1−β)=0.8 at a significance level of 0.05, ratio of controls to cases =1, hypothetical proportion of controls with exposure =9, and least extreme odds ratio (OR) to be detected =1.5 [54]. We initiated a population-based study at Tri-Service General Hospital (TSGH), a medical teaching hospital of the National Defense Medical Center in Taipei, Taiwan. This study was reviewed and approved by the Tri-Service General Hospital institutional ethical committee (TSGH-1-104-05-006). All enrolled study participants provided signed informed consent.

### Subjects

The case group was recruited from TSGH dialysis centers. All included patients were undergoing dialysis treatment and had been diagnosed with end-stage renal disease (ESRD). We recruited controls at the TSGH Health Management Center from patients participating in a check-up program beginning in March 2011. Control inclusion criteria were as follows: (1) estimated glomerular filtration rate (eGFR) of >60 ml/min/1.73 m^2^ as calculated using the MDRD equation; (2) no symptoms of kidney damage (e.g. proteinuria, hematuria); (3) no serious diseases (e.g. cancer), (4) able to provide a sufficient blood sample for genotyping. Patient demographic data, including age, sex, body mass index (BMI: kg/m^2^), history of hypertension, history of diabetes mellitus (DM), and smoking habits, were collected from medical records. Biochemistry laboratory values, such as total cholesterol, triglyceride, and creatinine levels, were collected from electronic health records. In total, 634 cases (296 men and 338 women) and 739 controls (298 men and 441 women) participated in this study up to July 2015.

### Genomic DNA extraction and genotyping

Genomic DNA was extracted from peripheral blood samples using standard proteinase K (Invitrogen, Carlsbad, CA, USA) digestion and phenol/chloroform extraction methods. AGTR1 A1166C polymorphisms were genotyped using the iPLEX Gold SNP genotyping method [55]. To validate results, at least 10% of samples were randomly selected for repeated genotyping.

### Case-control study statistical analysis

Continuous variables were evaluated using Student's *t* test and reported as means ± standard deviations (SDs). Genotypes and allelic frequencies were compared between dialysis patients and healthy controls using the χ^2^ test or Fisher's exact test as appropriate. Logistic regression was used to estimate ORs and 95% confidence intervals (CIs) as measures of the association with CKD risk. Allele type, co-dominant, and dominant/recessive models were used to calculate the association between genetic polymorphism and CKD risk. P<0.05 was considered significant. Statistical analyses were conducted using R software, version 3.3.1 (R Project for Statistical Computing, Vienna, Austria).

### Meta-analysis search methods and criteria for study consideration

The PRISMA checklist and Meta-analysis of Genetic Association Studies Checklist are described in Supplementary Table 1 [56]. This general population study compared CKD risk between individuals carrying the major (A) or minor (C) AGTR1 A1166C alleles. To identify relevant studies, we searched the PubMed and Cochrane Library databases for English-language articles using relevant words and medical subject headings that included all spellings of AGTR1 A1166C and CKD (Supplementary Table 2). We also manually scanned reference lists of identified trials and review articles to avoid missing relevant studies. We included all articles published from the dates of inception of these databases to December 2015.

All studies that assessed the association between AGTR1 A1166C and CKD risk were considered for inclusion using the following criteria: (1) cross-sectional surveys or case-control studies; (2) study population aged >18 years; (3) CKD defined according to the National Kidney Foundation as kidney damage by clinical diagnosis or a glomerular filtration rate <60 ml/min/1.73 m2; (4) inclusion of at least one control group with normal kidney function; and (5) detailed genotype information. Common genetic association study methods included co-dominant, allele, dominant, and recessive models, although some papers only presented significant results. We only included papers with detailed information about genotypes. Studies that investigated relationships between genetic polymorphisms and other kidney diseases (lupus nephritis, polycystic kidney disease, endemic nephropathy, or reflux nephropathy) were excluded. For manuscripts with incomplete published data, we attempted to contact the authors for further information.

### Data extraction and quality assessment

We recorded the first author's name, publication year, study population ethnicity, kidney functions of cases, definition of the case group, and case group population characteristics (mean age, proportion of male subjects, BMI, DM prevalence, hypertension prevalence, proportion of smokers, and AGTR1 A1166C genotype distribution). DM and hypertension were defined as plasma glucose >126 mg/dL and systolic blood pressure >140 mmHg, respectively. If the article did not report DM and hypertension prevalence, or the definitions did not match, we assumed normal plasma glucose level and systolic blood pressure distributions for calculation purposes.

Bias risk was assessed via the Cochrane Collaboration, as suggested by the Newcastle–Ottawa Quality Assessment Scale [57]. This tool focuses on the following factors: (1) selection of study population, (2) case and control group comparability, and (3) assessed exposure. Each study received a score between 0 and 9. We investigated the relationship between study quality and risk estimation.

### Meta-analysis statistical analysis

Population characteristics of each included study are presented as means or proportions as appropriate. Our meta-analysis examined the association between AGTR1 A1166C and CKD risk in each study using ORs with 95% CIs. The τ^2^ statistic, estimated according to the DerSimonian-Laird method, was used to assess heterogeneity, and a random-effects model was used to calculate weighed effect size. Three common genetic models (allele type, dominant, and recessive models) were used to calculate the association between AGTR1 A1166C and CKD risk.

Egger's regression and a funnel plot were used to test the symmetry of pooled results. The I^2^ value was calculated using the Cochrane Q test and used to quantify heterogeneity; an I^2^ value >50% indicated moderate to high heterogeneity. A meta-regression analysis of average summary values was used to explore the source of heterogeneity. According to our previous studies, the average summary value of a case group can be used to build a model and can facilitate interaction effect estimation [58]. An interaction effect is determined using the OR and defined as the ratio of ORs per unit. Possible moderators (race, study design, quality score, kidney function of case, sex, age, BMI, hypertension, DM, and smoking) were tested to explore heterogeneity.

P<0.05 was considered significant. However, because of multiple comparison correction, P<0.05/10 was considered significant for meta-regression analyses. Statistical analyses were conducted using the “metafor” and “meta” R version 3.3.1 packages.

## SUPPLEMENTARY MATERIALS TABLES








