# Supplementary material for: Association between angiotensin II receptor type 1 A1166C polymorphism and chronic kidney disease
Source: Oncotarget. 2018 Feb 12;9(18):14444–55. doi: 10.18632/oncotarget.24469 (PMC5865681; doi:10.18632/oncotarget.24469)
Supplement: Supplementary file 1 [file oncotarget-09-14444-s001.pdf]

Association between angiotensin II receptor type 1 A1166C polymorphism and chronic kidney disease

SUPPLEMENTARY MATERIALS

Supplementary Table 1: PRISMA 2009 Checklist.

See Supplementary File 1

Supplementary Table 2: Meta-analysis search methods and criteria for study consideration.

See Supplementary File 2

Supplementary Table 3: Summary of studies included in the meta-analysis.

See Supplementary File 3
