# Supplementary material for: Association between angiotensin II receptor type 1 A1166C polymorphism and chronic kidney disease
Source: Oncotarget. 2018 Feb 12;9(18):14444–55. doi: 10.18632/oncotarget.24469 (PMC5865681; doi:10.18632/oncotarget.24469)
Supplement: Supplementary file 3 [file oncotarget-09-14444-s003.docx]

**S2 Table. Search strategies and detailed records.**

| **Relevant text of AGTR1 A1166C**   1. Receptor, Angiotensin II Type 1 2. Angiotensin Type 1 Receptor 3. Angiotensin AT1 Receptor 4. AT1 Receptor, Angiotensin 5. Receptor, Angiotensin AT1 6. Angiotensin II Type 1 Receptor 7. AT1 Receptor, Angiotensin 8. AGTR1 9. A1166C 10. rs5186 11. ((1 or 2 or 3 or 4 or 5 or 6 or 7 or 8) and 9) or 10 | **Relevant text of chronic kidney disease**   1. End-Stage Kidney Disease 2. End-Stage Renal Disease 3. End-Stage Renal Failure 4. ESRD 5. ESRF 6. ESKD 7. Chronic kidney disease 8. CKD 9. dialysis 10. eGFR 11. estimated Glomerular filtration rate 12. nephropathy 13. 12 or 13 or 14 or 15 or 16 or 17 or 18 or 19 or 20 or 21 or 22 or 23   **Combined (Final strategy)**  11 and 24 |
| --- | --- |

Web sites and uniform resource locator:

**PubMed**: <http://www.ncbi.nlm.nih.gov/pubmed>

**Cochrane Library**: <http://www.thecochranelibrary.com>

**Records from PUBMED**

Unrelated records (wrong outcome) [1-39]

Duplicated samples [40-43]

Paper didn’t provide detailed genotyping data [44-47]

Included studies [48-68]

**Records from Cochrane Library:**

Duplicated records [25]

Unrelated records (wrong outcome) [69]

**Records from manually scan:**

Unrelated records (wrong outcome) [70-73]

Duplicated samples [74, 75]

Included studies [76, 77]

**Reference**

1. Katsuya T, Morishita R. Gene polymorphism of angiotensin II type 1 and type 2 receptors. Current pharmaceutical design. 2013;19(17):2996-3001. Epub 2012/11/28. PubMed PMID: 23176211.

2. Kaczmarczyk M, Kuprjanowicz A, Loniewska B, Goracy I, Taryma-Lesniak O, Skonieczna-Zydecka K, et al. Epistatic interaction between common AGT G(-6)A (rs5051) and AGTR1 A1166C (rs5186) variants contributes to variation in kidney size at birth. Gene. 2015;572(1):72-8. Epub 2015/07/05. doi: 10.1016/j.gene.2015.06.071. PubMed PMID: 26142106.

3. Deckers IA, van den Brandt PA, van Engeland M, van Schooten FJ, Godschalk RW, Keszei AP, et al. Polymorphisms in genes of the renin-angiotensin-aldosterone system and renal cell cancer risk: interplay with hypertension and intakes of sodium, potassium and fluid. International journal of cancer Journal international du cancer. 2015;136(5):1104-16. Epub 2014/07/01. doi: 10.1002/ijc.29060. PubMed PMID: 24978482.

4. Raizada V, Skipper B, Luo W, Garza L, Hines CW, Harford AA, et al. Renin-angiotensin polymorphisms and QTc interval prolongation in end-stage renal disease. Kidney Int. 2005;68(3):1186-9. Epub 2005/08/18. doi: 10.1111/j.1523-1755.2005.00510.x. PubMed PMID: 16105049.

5. Chudek J, Szotowska M, Karkoszka H, Verbeke F, Trautsolt W, Gumprecht J, et al. Genotypes of renin-angiotensin system and plasma adiponectin concentration in kidney transplant patients. Annals of transplantation : quarterly of the Polish Transplantation Society. 2013;18:593-603. Epub 2013/11/05. doi: 10.12659/aot.884022. PubMed PMID: 24185422.

6. Zivkovic M, Stankovic A, Alavantic D. AT1 receptor A1166C and AT2 receptor -1332A/G gene polymorphisms: efficient genotyping by single-tube PCR. Journal of clinical laboratory analysis. 2005;19(2):84-6. Epub 2005/03/10. doi: 10.1002/jcla.20058. PubMed PMID: 15756705.

7. Lee KB, Kim UK. Angiotensinogen and angiotensin II type 1 receptor gene polymorphism in patients with autosomal dominant polycystic kidney disease: effect on hypertension and ESRD. Yonsei medical journal. 2003;44(4):641-7. Epub 2003/09/02. PubMed PMID: 12950120.

8. Boger CA, Gotz AK, Kruger B, Hosl M, Schmitz G, Riegger GA, et al. Effect of genetic variation on therapy with angiotensin converting enzyme inhibitors or angiotensin receptor blockers in dialysis patients. European journal of medical research. 2005;10(4):161-8. Epub 2005/06/11. PubMed PMID: 15946912.

9. Kujawa-Szewieczek A, Kolonko A, Kocierz M, Szotowska M, Trusolt W, Karkoszka H, et al. Association between gene polymorphisms of the components of the renin-angiotensin-aldosteron system, graft function, and the prevalence of hypertension, anemia, and erythrocytosis after kidney transplantation. Transplantation proceedings. 2011;43(8):2957-63. Epub 2011/10/15. doi: 10.1016/j.transproceed.2011.07.016. PubMed PMID: 21996200.

10. Konoshita T, Miyagi K, Onoe T, Katano K, Mutoh H, Nomura H, et al. Effect of ACE gene polymorphism on age at renal death in polycystic kidney disease in Japan. Am J Kidney Dis. 2001;37(1):113-8. Epub 2001/01/03. PubMed PMID: 11136175.

11. van Geel PP, Pinto YM, Voors AA, Buikema H, Oosterga M, Crijns HJ, et al. Angiotensin II type 1 receptor A1166C gene polymorphism is associated with an increased response to angiotensin II in human arteries. Hypertension. 2000;35(3):717-21. Epub 2000/03/18. PubMed PMID: 10720584.

12. Nobilis A, Kocsis I, Toth-Heyn P, Treszl A, Schuler A, Tulassay T, et al. Variance of ACE and AT1 receptor gene does not influence the risk of neonatal acute renal failure. Pediatric nephrology (Berlin, Germany). 2001;16(12):1063-6. Epub 2002/01/17. doi: 10.1007/s004670100028. PubMed PMID: 11793101.

13. Siekierka-Harreis M, Kuhr N, Willers R, Ivens K, Grabensee B, Mondry A, et al. Impact of genetic polymorphisms of the renin-angiotensin system and of non-genetic factors on kidney transplant function--a single-center experience. Clinical transplantation. 2009;23(5):606-15. Epub 2009/08/18. doi: 10.1111/j.1399-0012.2009.01033.x. PubMed PMID: 19681973.

14. Zhang G, Wang H, Wang F, Yu L, Yang X, Meng J, et al. Gene polymorphisms of the renin-angiotensin-aldosterone system and angiotensin II type 1-receptor activating antibodies in renal rejection. Tohoku J Exp Med. 2007;213(3):203-14. Epub 2007/11/07. PubMed PMID: 17984617.

15. Manea SA, Robciuc A, Guja C, Heltianu C. Identification of gene variants in NOS3, ET-1 and RAS that confer risk and protection against microangiopathy in type 2 diabetic obese subjects. Biochemical and biophysical research communications. 2011;407(3):486-90. Epub 2011/03/17. doi: 10.1016/j.bbrc.2011.03.043. PubMed PMID: 21406182.

16. Lee YJ, Jang HR, Kim SG, Chae DW, Do JY, Lee JE, et al. Renoprotective efficacy of valsartan in chronic non-diabetic proteinuric nephropathies with renin-angiotensin system gene polymorphisms. Nephrology (Carlton). 2011;16(5):502-10. Epub 2011/02/10. doi: 10.1111/j.1440-1797.2011.01448.x. PubMed PMID: 21303424.

17. Yin X, Li H, Xuan J, Chen Y, Li L, Dong X. [AGTR1 A1166C polymorphism is associated with risk of diabetic nephropathy]. Zhejiang da xue xue bao Yi xue ban = Journal of Zhejiang University Medical sciences. 2013;42(1):45-51. Epub 2013/03/19. PubMed PMID: 23505107.

18. Buraczynska M, Jozwiak L, Spasiewicz D, Nowicka T, Ksiazek A. [Renin-angiotensin system genes in chronic glomerulonephritis]. Polskie Archiwum Medycyny Wewnetrznej. 2001;105(6):455-60. Epub 2002/02/28. PubMed PMID: 11865575.

19. Buraczynska M, Ksiazek P, Lopatynski J, Spasiewicz D, Nowicka T, Ksiazek A. [Association of the renin-angiotensin system gene polymorphism with nephropathy in type II diabetes]. Polskie Archiwum Medycyny Wewnetrznej. 2002;108(2):725-30. Epub 2002/12/13. PubMed PMID: 12476891.

20. Zhou TB, Yin SS, Jiang ZP. Association of angiotensin II type-1 receptor A1166C gene polymorphism with the susceptibility of end-stage renal disease. Journal of receptor and signal transduction research. 2013;33(5):325-31. Epub 2013/08/27. doi: 10.3109/10799893.2013.828071. PubMed PMID: 23971628.

21. Mao S, Huang S. Lack of association of angiotensin II type 1 receptor A1166C gene polymorphism with the risk of end-stage renal disease. Renal failure. 2013;35(9):1295-301. Epub 2013/08/02. doi: 10.3109/0886022x.2013.820663. PubMed PMID: 23902432.

22. Zhou TB, Jiang ZP, Zhou JF, Zhang YM. Association of angiotensin II type-1 receptor A1166C gene polymorphism with the susceptibility of immunoglobulin A nephropathy. Renal failure. 2015;37(3):359-62. Epub 2015/01/15. doi: 10.3109/0886022x.2014.1000800. PubMed PMID: 25585948.

23. Braliou GG, Grigoriadou AM, Kontou PI, Bagos PG. The role of genetic polymorphisms of the Renin-Angiotensin System in renal diseases: A meta-analysis. Computational and structural biotechnology journal. 2014;10(16):1-7. Epub 2014/09/12. doi: 10.1016/j.csbj.2014.05.006. PubMed PMID: 25210592; PubMed Central PMCID: PMCPmc4151998.

24. Ding W, Wang F, Fang Q, Zhang M, Chen J, Gu Y. Association between two genetic polymorphisms of the renin-angiotensin-aldosterone system and diabetic nephropathy: a meta-analysis. Mol Biol Rep. 2012;39(2):1293-303. doi: 10.1007/s11033-011-0862-7. PubMed PMID: 21607620.

25. Buraczynska M, Grzebalska A, Spasiewicz D, Orlowska G, Ksiazek A. Genetic polymorphisms of renin-angiotensin system and progression of interstitial nephritis. Annales Universitatis Mariae Curie-Sklodowska Sectio D: Medicina. 2002;57(2):330-6. Epub 2003/08/06. PubMed PMID: 12898858.

26. Kostic M, Stankovic A, Zivkovic M, Peco-Antic A, Jovanovic O, Alavantic D, et al. ACE and AT1 receptor gene polymorphisms and renal scarring in urinary bladder dysfunction. Pediatric nephrology (Berlin, Germany). 2004;19(8):853-7. Epub 2004/06/05. doi: 10.1007/s00467-004-1511-3. PubMed PMID: 15179569.

27. Pardo R, Malaga S, Coto E, Navarro M, Alvarez V, Espinosa L, et al. Renin-angiotensin system polymorphisms and renal scarring. Pediatric nephrology (Berlin, Germany). 2003;18(2):110-4. Epub 2003/02/13. doi: 10.1007/s00467-002-1031-y. PubMed PMID: 12579398.

28. Ece A, Tekes S, Gurkan F, Bilici M, Budak T. Polymorphisms of the angiotensin converting enzyme and angiotensin II type 1 receptor genes and renal scarring in non-uropathic children with recurrent urinary tract infection. Nephrology (Carlton). 2005;10(4):377-81. Epub 2005/08/20. doi: 10.1111/j.1440-1797.2005.00430.x. PubMed PMID: 16109085.

29. Liu KP, Lin CY, Chen HJ, Wei CF, Lee-Chen GJ. Renin-angiotensin system polymorphisms in Taiwanese primary vesicoureteral reflux. Pediatric nephrology (Berlin, Germany). 2004;19(6):594-601. Epub 2004/03/27. doi: 10.1007/s00467-003-1379-7. PubMed PMID: 15045574.

30. Papp F, Friedman AL, Bereczki C, Haszon I, Kiss E, Endreffy E, et al. Renin-angiotensin gene polymorphism in children with uremia and essential hypertension. Pediatric nephrology (Berlin, Germany). 2003;18(2):150-4. Epub 2003/02/13. doi: 10.1007/s00467-002-1032-x. PubMed PMID: 12579405.

31. Filler G, Yang F, Martin A, Stolpe J, Neumayer H-H, Hocher B. Renin angiotensin system gene polymorphisms in pediatric renal transplant recipients. Pediatric Transplantation. 2001;5(3):166-73. doi: 10.1034/j.1399-3046.2001.00053.x.

32. Maruyama K, Yoshida M, Nishio H, Shirakawa T, Kawamura T, Tanaka R, et al. Polymorphisms of renin-angiotensin system genes in childhood IgA nephropathy. Pediatric nephrology (Berlin, Germany). 2001;16(4):350-5. Epub 2001/05/17. PubMed PMID: 11354780.

33. Gallego PH, Shephard N, Bulsara MK, van Bockxmeer FM, Powell BL, Beilby JP, et al. Angiotensinogen gene T235 variant: a marker for the development of persistent microalbuminuria in children and adolescents with type 1 diabetes mellitus. J Diabetes Complications. 2008;22(3):191-8. doi: 10.1016/j.jdiacomp.2007.03.003. PubMed PMID: 18413222.

34. Yoshida H, Kuriyama S, Atsumi Y, Tomonari H, Mitarai T, Hamaguchi A, et al. Angiotensin I converting enzyme gene polymorphism in non-insulin dependent diabetes mellitus. Kidney Int. 1996;50(2):657-64. Epub 1996/08/01. PubMed PMID: 8840299.

35. Frimat L, Philippe C, Maghakian MN, Jonveaux P, Hurault de Ligny B, Guillemin F, et al. Polymorphism of angiotensin converting enzyme, angiotensinogen, and angiotensin II type 1 receptor genes and end-stage renal failure in IgA nephropathy: IGARAS--a study of 274 Men. J Am Soc Nephrol. 2000;11(11):2062-7. Epub 2000/10/29. PubMed PMID: 11053482.

36. Hunley TE, Julian BA, Phillips JA, 3rd, Summar ML, Yoshida H, Horn RG, et al. Angiotensin converting enzyme gene polymorphism: potential silencer motif and impact on progression in IgA nephropathy. Kidney Int. 1996;49(2):571-7. Epub 1996/02/01. PubMed PMID: 8821846.

37. Coll E, Campos B, Gonzalez-Nunez D, Botey A, Poch E. Association between the A1166C polymorphism of the angiotensin II receptor type 1 and progression of chronic renal insufficiency. Journal of nephrology. 2003;16(3):357-64. Epub 2003/07/02. PubMed PMID: 12832734.

38. Hsu CC, Bray MS, Kao WH, Pankow JS, Boerwinkle E, Coresh J. Genetic variation of the renin-angiotensin system and chronic kidney disease progression in black individuals in the atherosclerosis risk in communities study. J Am Soc Nephrol. 2006;17(2):504-12. Epub 2006/01/07. doi: 10.1681/asn.2005050468. PubMed PMID: 16396964.

39. Lee YT, Chiu HC, Huang CT, Su HM, Wang CL, Lin TH, et al. The A1166C polymorphism of angiotensin II Type 1 receptor as a predictor of renal function decline over 4 years follow-up in an apparently healthy Chinese population. Clinical nephrology. 2009;72(6):457-67. Epub 2009/12/04. PubMed PMID: 19954723.

40. Su SL, Lu KC, Lin YF, Hsu YJ, Lee PY, Yang HY, et al. Gene polymorphisms of angiotensin-converting enzyme and angiotensin II type 1 receptor among chronic kidney disease patients in a Chinese population. J Renin Angiotensin Aldosterone Syst. 2012;13(1):148-54. Epub 2011/12/08. doi: 10.1177/1470320311430989. PubMed PMID: 22147663.

41. Su SL, Yang HY, Wu CC, Lee HS, Lin YF, Hsu CA, et al. Gene-gene interactions in renin-angiotensin-aldosterone system contributes to end-stage renal disease susceptibility in a Han Chinese population. ScientificWorldJournal. 2014;2014:169798. doi: 10.1155/2014/169798. PubMed PMID: 24977181; PubMed Central PMCID: PMCPMC4003748.

42. Buraczynska M, Ksiazek P, Zaluska W, Spasiewicz D, Nowicka T, Ksiazek A. Angiotensin II type 1 receptor gene polymorphism in end-stage renal disease. Nephron. 2002;92(1):51-5. Epub 2002/08/21. PubMed PMID: 12187084.

43. Ahluwalia TS, Ahuja M, Rai TS, Kohli HS, Bhansali A, Sud K, et al. ACE variants interact with the RAS pathway to confer risk and protection against type 2 diabetic nephropathy. DNA and cell biology. 2009;28(3):141-50. Epub 2008/12/26. doi: 10.1089/dna.2008.0810. PubMed PMID: 19108684.

44. Anbazhagan K, Sampathkumar K, Ramakrishnan M, Gomathi P, Gomathi S, Selvam GS. Analysis of polymorphism in renin angiotensin system and other related genes in South Indian chronic kidney disease patients. Clinica chimica acta; international journal of clinical chemistry. 2009;406(1-2):108-12. Epub 2009/06/13. doi: 10.1016/j.cca.2009.06.003. PubMed PMID: 19520069.

45. Bantis C, Ivens K, Kreusser W, Koch M, Klein-Vehne N, Grabensee B, et al. Influence of genetic polymorphisms of the renin-angiotensin system on IgA nephropathy. Am J Nephrol. 2004;24(2):258-67. Epub 2004/03/20. doi: 10.1159/000077398. PubMed PMID: 15031629.

46. Tomino Y, Makita Y, Shike T, Gohda T, Haneda M, Kikkawa R, et al. Relationship between polymorphism in the angiotensinogen, angiotensin-converting enzyme or angiotensin II receptor and renal progression in Japanese NIDDM patients. Nephron. 1999;82(2):139-44.

47. Chang HR, Cheng CH, Shu KH, Chen CH, Lian JD, Wu MY. Study of the polymorphism of angiotensinogen, anigiotensin-converting enzyme and angiotensin receptor in type II diabetes with end-stage renal disease in Taiwan. Journal of the Chinese Medical Association : JCMA. 2003;66(1):51-6. Epub 2003/05/06. PubMed PMID: 12728975.

48. Buraczynska M, Ksiazek P, Drop A, Zaluska W, Spasiewicz D, Ksiazek A. Genetic polymorphisms of the renin-angiotensin system in end-stage renal disease. Nephrol Dial Transplant. 2006;21(4):979-83. doi: 10.1093/ndt/gfk012. PubMed PMID: 16384824.

49. Chen WJ, Huang YL, Shiue HS, Chen TW, Lin YF, Huang CY, et al. Renin-angiotensin-aldosterone system related gene polymorphisms and urinary total arsenic is related to chronic kidney disease. Toxicology and applied pharmacology. 2014;279(2):95-102. Epub 2014/06/08. doi: 10.1016/j.taap.2014.05.011. PubMed PMID: 24907556.

50. Fabris B, Bortoletto M, Candido R, Barbone F, Cattin MR, Calci M, et al. Genetic polymorphisms of the renin-angiotensin-aldosterone system and renal insufficiency in essential hypertension. J Hypertens. 2005;23(2):309-16. Epub 2005/01/22. PubMed PMID: 15662219.

51. Fradin S, Goulet-Salmon B, Chantepie M, Grandhomme F, Morello R, Jauzac P, et al. Relationship between polymorphisms in the renin-angiotensin system and nephropathy in type 2 diabetic patients. Diabetes & metabolism. 2002;28(1):27-32. Epub 2002/04/09. PubMed PMID: 11938025.

52. Gao J, Yu QL, Fu RG, Wei LT, Wang M, Dong FM, et al. Lack of Association Between Polymorphisms in AGT and ATR1 and IgA Nephropathy in a Chinese Population. Genetic testing and molecular biomarkers. 2015;19(12):710-3. Epub 2015/11/21. doi: 10.1089/gtmb.2015.0167. PubMed PMID: 26588355.

53. Huang HD, Lin FJ, Li XJ, Wang LR, Jiang GR. Genetic polymorphisms of the renin-angiotensin-aldosterone system in Chinese patients with end-stage renal disease secondary to IgA nephropathy. Chinese medical journal. 2010;123(22):3238-42. Epub 2010/12/18. PubMed PMID: 21163122.

54. Kim SM, Chin HJ, Oh YK, Kim YS, Kim S, Lim CS. Blood pressure-related genes and the progression of IgA nephropathy. Nephron Clinical practice. 2009;113(4):c301-8. Epub 2009/09/05. doi: 10.1159/000235948. PubMed PMID: 19729965.

55. Losito A, Kalidas K, Santoni S, Ceccarelli L, Jeffery S. Polymorphism of renin-angiotensin system genes in dialysis patients--association with cerebrovascular disease. Nephrol Dial Transplant. 2002;17(12):2184-8. Epub 2002/11/28. PubMed PMID: 12454231.

56. Marre M, Jeunemaitre X, Gallois Y, Rodier M, Chatellier G, Sert C, et al. Contribution of genetic polymorphism in the renin-angiotensin system to the development of renal complications in insulin-dependent diabetes: Genetique de la Nephropathie Diabetique (GENEDIAB) study group. The Journal of clinical investigation. 1997;99(7):1585-95. Epub 1997/04/01. doi: 10.1172/jci119321. PubMed PMID: 9120002; PubMed Central PMCID: PMCPmc507978.

57. Osawa N, Koya D, Araki S, Uzu T, Tsunoda T, Kashiwagi A, et al. Combinational effect of genes for the renin-angiotensin system in conferring susceptibility to diabetic nephropathy. J Hum Genet. 2007;52(2):143-51. doi: 10.1007/s10038-006-0090-5. PubMed PMID: 17143591.

58. Pei Y, Scholey J, Thai K, Suzuki M, Cattran D. Association of angiotensinogen gene T235 variant with progression of immunoglobin A nephropathy in Caucasian patients. The Journal of clinical investigation. 1997;100(4):814-20. Epub 1997/08/15. doi: 10.1172/jci119596. PubMed PMID: 9259580; PubMed Central PMCID: PMCPmc508253.

59. Stratta P, Bermond F, Guarrera S, Canavese C, Carturan S, Dall'Omo A, et al. Interaction between gene polymorphisms of nitric oxide synthase and renin-angiotensin system in the progression of membranous glomerulonephritis. Nephrol Dial Transplant. 2004;19(3):587-95. Epub 2004/02/10. PubMed PMID: 14767013.

60. van Ittersum FJ, de Man AM, Thijssen S, de Knijff P, Slagboom E, Smulders Y, Tarnow L, Donker AJ, Bilo HJ, Stehouwer CD. Genetic polymorphisms of the renin-angiotensin system and complications of insulin-dependent diabetes mellitus. Nephrol Dial Transplant. 2000; 15:1000-7.

61. Woo KT, Lau YK, Choong LH, Zhao Y, Tan HB, Fook-Chong S, et al. Polymorphism of renin-angiotensin system genes in IgA nephropathy. Nephrology (Carlton). 2004;9(5):304-9. Epub 2004/10/27. doi: 10.1111/j.1440-1797.2004.00291.x. PubMed PMID: 15504143.

62. Wu S, Xiang K, Zheng T, Sun D, Weng Q, Zhao H, et al. Relationship between the renin-angiotensin system genes and diabetic nephropathy in the Chinese. Chinese medical journal. 2000;113(5):437-41. Epub 2002/01/05. PubMed PMID: 11776100.

63. Zsom M, Fulop T, Zsom L, Barath A, Maroti Z, Endreffy E. Genetic polymorphisms and the risk of progressive renal failure in elderly Hungarian patients. Hemodialysis international International Symposium on Home Hemodialysis. 2011;15(4):501-8. Epub 2011/11/25. doi: 10.1111/j.1542-4758.2011.00593.x. PubMed PMID: 22111818.

64. Chowdhury TA, Dyer PH, Kumar S, Gough SC, Gibson SP, Rowe BR, et al. Lack of association of angiotensin II type 1 receptor gene polymorphism with diabetic nephropathy in insulin-dependent diabetes mellitus. Diabetic medicine : a journal of the British Diabetic Association. 1997;14(10):837-40. Epub 1997/11/26. doi: 10.1002/(sici)1096-9136(199710)14:10<837::aid-dia463>3.0.co;2-v. PubMed PMID: 9371475.

65. Hanna MO, Shahin RM, Meshaal SS, Kostandi IF. Susceptibility and progression of end stage renal disease are not associated with angiotensin II type 1 receptor gene polymorphism. Journal of receptor and signal transduction research. 2015;35(5):381-5. Epub 2014/10/16. doi: 10.3109/10799893.2014.956757. PubMed PMID: 25316403.

66. Mollsten A, Vionnet N, Forsblom C, Parkkonen M, Tarnow L, Hadjadj S, et al. A polymorphism in the angiotensin II type 1 receptor gene has different effects on the risk of diabetic nephropathy in men and women. Molecular genetics and metabolism. 2011;103(1):66-70. Epub 2011/02/15. doi: 10.1016/j.ymgme.2011.01.004. PubMed PMID: 21316998.

67. Moradi M, Rahimi Z, Amiri S, Rahimi Z, Vessal M, Nasri H. AT1R A1166C variants in patients with type 2 diabetes mellitus and diabetic nephropathy. Journal of nephropathology. 2015;4(3):69-76. Epub 2015/08/28. doi: 10.12860/jnp.2015.14. PubMed PMID: 26310144; PubMed Central PMCID: PMCPmc4544557.

68. Shah VN, Cheema BS, Sharma R, Khullar M, Kohli HS, Ahluwalia TS, et al. ACACbeta gene (rs2268388) and AGTR1 gene (rs5186) polymorphism and the risk of nephropathy in Asian Indian patients with type 2 diabetes. Molecular and cellular biochemistry. 2013;372(1-2):191-8. Epub 2012/10/20. doi: 10.1007/s11010-012-1460-2. PubMed PMID: 23081748.

69. Isbir SC, Tekeli A, Ergen A, Yilmaz H, Ak K, Civelek A, et al. Genetic polymorphisms contribute to acute kidney injury after coronary artery bypass grafting. The heart surgery forum. 2007;10(6):E439-44. Epub 2007/10/09. doi: 10.1532/hsf98.20071117. PubMed PMID: 17921131.

70. Tabel Y, Berdeli A, Mir S, Serdaroglu E, Yilmaz E. Effects of genetic polymorphisms of the renin-angiotensin system in children with nephrotic syndrome. J Renin Angiotensin Aldosterone Syst. 2005;6(3):138-44. doi: 10.3317/jraas.2005.020. PubMed PMID: 16525944.

71. Elshamaa MF, Sabry SM, Bazaraa HM, Koura HM, Elghoroury EA, Kantoush NA, et al. Genetic polymorphism of ACE and the angiotensin II type1 receptor genes in children with chronic kidney disease. J Inflamm (Lond). 2011;8(1):20. doi: 10.1186/1476-9255-8-20. PubMed PMID: 21859496; PubMed Central PMCID: PMCPMC3167745.

72. Ayed K, Ayed-Jendoubi S, Ben Abdallah T, Bardi R, Gorgi Y, Sfar I, et al. Polymorphism of the renin-angiotensin-aldosterone system in patients with chronic allograft dysfunction. Transpl Immunol. 2006;15(4):303-9. doi: 10.1016/j.trim.2005.09.011. PubMed PMID: 16635753.

73. Gumprecht J, Zychma MJ, Grzeszczak W, Zukowska-Szczechowska E. Angiotensin I-converting enzyme gene insertion/deletion and angiotensinogen M235T polymorphisms: risk of chronic renal failure. End-Stage Renal Disease Study Group. Kidney Int. 2000;58(2):513-9. Epub 2000/08/01. doi: 10.1046/j.1523-1755.2000.00197.x. PubMed PMID: 10916074.

74. Lau YK, Woo KT, Choong HL, Zhao Y, Tan HB, Chong SM, et al. Renin-angiotensin system gene polymorphisms: its impact on IgAN and its progression to end-stage renal failure among Chinese in Singapore. Nephron Physiol. 2004;97(1):p1-8. doi: 10.1159/000077596. PubMed PMID: 15153745.

75. Mollsten A, Kockum I, Svensson M, Rudberg S, Ugarph-Morawski A, Brismar K, et al. The effect of polymorphisms in the renin-angiotensin-aldosterone system on diabetic nephropathy risk. J Diabetes Complications. 2008;22(6):377-83. doi: 10.1016/j.jdiacomp.2007.06.005. PubMed PMID: 18413189.

76. Prasad P, Tiwari AK, Kumar KM, Ammini AC, Gupta A, Gupta R, et al. Chronic renal insufficiency among Asian Indians with type 2 diabetes: I. Role of RAAS gene polymorphisms. BMC Med Genet. 2006;7:42. doi: 10.1186/1471-2350-7-42. PubMed PMID: 16672053; PubMed Central PMCID: PMCPMC1479320.

77. El-Essawy AB, Berthoux P, Cécillon S, C.Deprèle, Thibaudin D, Filippis JPD, et al. Hypertension after renal transplantation and polymorphism of genes involved in essential hypertension: ACE, AGT, AT1R and ecNOS. Clinical Nephrology. 2002;57(03):192-200. doi: 10.5414/cnp57192.
