# Supplementary material for: Association between angiotensin II receptor type 1 A1166C polymorphism and chronic kidney disease
Source: Oncotarget. 2018 Feb 12;9(18):14444–55. doi: 10.18632/oncotarget.24469 (PMC5865681; doi:10.18632/oncotarget.24469)
Supplement: Supplementary file 4 [file oncotarget-09-14444-s004.pdf]

Supplementary Table 3. Summary of studies included in the meta-analysis.

Author	Year	Country	Ethnicity	Study design[a]	CKD type[b]	Kidney function of case[c]	Definition of case group[d]
This Study		Taiwan	Asian	CC	Mixed	ESRD	Dialysis patient
Gao [1]	2015	China	Asian	CC	IgAN	non-ESRD	histologically biopsy
Moradi [2]	2015	Iran	Caucasian	CC	DN	non-ESRD	ACR> 30 mg/g
Chen [3]	2014	Taiwan	Asian	CC	Mixed	non-ESRD	eGFR < 60 ml/min/1.73 m2
Hanna [4]	2014	Egypt	Caucasian	CC	Mixed	ESRD	HD
Shah [5]	2013	India	Asian	CS	DN	non-ESRD	ACR>300mg/g or UAE >500mg/24h
Mollsten [6]	2011	Denmark	Caucasian	CS	DN	non-ESRD	AER>200ug/min or ACR>300mg/g or HD
Zsom [7]	2011	Hungary	Caucasian	CC	Mixed	non-ESRD	histologically biopsy & eGFR < 60 ml/min/1.73 m[illegible]
Huang [8]	2010	China	Asian	CC	IgAN	non-ESRD	histologically biopsy
Kim [9]	2009	Korea	Asian	CC	IgAN	non-ESRD	histologically biopsy
Buraczynska [10]	2006	Poland	Caucasian	CC	Mixed	ESRD	HD
Prasad [11]	2006	India	Asian	CS	DN	non-ESRD	SCr > 1.5 mg/dL or AER>200mg/L or RTN
Fabris [12]	2005	Italy	Caucasian	CC	HN	non-ESRD	SCr > 1.5 mg/dL or CT
Stratta [13]	2004	Italy	Caucasian	CC	GN	non-ESRD	CT &s-CR<1.5mg/dL
Woo [14]	2004	Singapore	Asian	CC	IgAN	non-ESRD	CT & UAE >1g/day
El-Essawy [15]	2002	France	Caucasian	CS	Mixed	ESRD	RRT
Fradin [16]	2002	France	Caucasian	CS	DN	non-ESRD	UAE >30 mg/day or > 20 μg/min
Losito [17]	2002	United Kingdom	Caucasian	CC	Mixed	ESRD	HD
Thomas [18]	2001	Hong Kong	Asian	CS	DN	non-ESRD	UAE > 20 μg/min
van Ittersum [19]	2000	Netherlands	Caucasian	CS	DN	non-ESRD	UAE >30 mg/day
Wu [20]	2000	China	Asian	CS	DN	non-ESRD	no description
Chowdhury [21]	1997	United Kingdom	Caucasian	CC	DN	non-ESRD	UAE >3 times in 6 months
Marre [22]	1997	France	Caucasian	CS	DN	non-ESRD	UAE > 30 mg/day
Pei [23]	1997	Canada	Caucasian	CC	IgAN	non-ESRD	histologically biopsy

Author	year	quality	age	sex	BMI	DM	HT	smoke	AA_case	AC_case	CC_case	AA_control	AC_control	CC_cont[illegible]

This Study		5	64.5	46.7	22.4		57.8	21.1	560	67	7	656	72	10
Gao [1]	2015	2	32	65.2		100	49.3		3	42	306	0	38	272
Moradi [2]	2015	7	56.3		27.4	100	42.1		71	21	2	28	13	0
Chen[3]	2014	6	65.1	61.4		39.1	55.8	27.5	208	25	0	407	42	0
Hanna[4]	2014	5	37.9	64		26	22		37	12	1	30	13	1
Shah(1)[5]	2013	6	60.1	39	27.8	100	59.5		104	112	24	131	119	5
Shah(2)[5]	2013	6	55.7	62	25.6	100	41.4		112	122	26	109	101	4
Shah(3)[5]	2013	6	60.5	55	24.5	100	56.8		39	45	12	50	40	2
Mollsten(1)[6]	2011	4	43	100		100	58	32.6	152	124	25	98	81	21
Mollsten(2)[6]	2011	4	40.4	0		100	50.1	45.3	85	63	19	105	96	14
Mollsten(3)[6]	2011	4	43	100		100	58	32.6	391	195	16	100	55	8
Mollsten(4)[6]	2011	4	40.4	0		100	50.1	45.3	261	145	13	184	63	6
Mollsten(5)[6]	2011	4	43	100		100	58	32.6	139	96	33	59	50	16
Mollsten(6)[6]	2011	4	40.4	0		100	50.1	45.3	114	80	17	69	54	10
Mollsten(7)[6]	2011	4	43	100		100	58	32.6	42	20	6	45	42	3
Mollsten(8)[6]	2011	4	40.4	0		100	50.1	45.3	58	29	3	60	43	9
Zsom[7]	2011	5	66	57.6					167	125	16	100	88	12
Huang[8]	2010	4	40	0.5			53.8		113	17	0	100	20	0
Kim(1)[9]	2009	5	32.4	100			55.6		114	16	0	132	10	0
Kim(2)[9]	2009	5	35	0			62.5		100	8	0	144	14	0
Buraczynska[10]	2006	5	51	55.7		19	78.2		346	322	77	322	182	16
Prasad[11]	2006	4	57	33.2		100			169	25	2	194	29	2
Fabris[12]	2005	5	60	77.9	25.9	0	100		40	42	4	106	59	7
Stratta[13]	2004	6	50	66.7			39.3		62	43	12	90	67	14
Woo[14]	2004	4	43	47.5			36.4		110	7	1	84	10	0
El-Essawy[15]	2002	5	42.2	33.3					150	125	19	101	63	17
Fradin[16]	2002	5	57	53	31.5	100			74	31	12	61	52	5
Losito[17]	2002	5	67	60.6	24.9	13.7	63.1		72	72	16	91	64	14
Thomas(1)[18]	2001	5	43.6	30.8	27.5	0	100		24	2	0	64	8	0

Thomas(2)[18]	2001	5	50.5	43	26.1	100	62.1		139	11	0	232	23	0
van Ittersum[19]	2000	5	55	59.4		100	79.7	33.8	30	31	8	70	100	19
Wu[20]	2000	5	60	54.9		100			56	15	0	33	8	0
Chowdhury[21]	1997	4	40	51.9		100	98		116	137	11	69	59	8
Marre[22]	1997	7	43	57.3	23.5	100	57.1		177	141	19	75	64	14
Pei[23]	1997	5							82	73	13	56	37	7

[a]: CC, case control study; CS, cross sectional study.

[b]: IgAN, IgA nephropathy; DN, diabetic nephropathy; GN, glomerulonephritis; Mixed, combine.

[c]: ESRD, only ESRD patients; non-ESRD, not only ESRD patients.

[d]: ACR, Albumin creatinine ratio; AER, Albumin excretion rate; UAE, urinary albumin excretion rate; eGFR, estimated glomerular filtration rate; HD, hemodialysis; CCr, creatinine clearance; RRT, renal replacement therapy; CT, computed tomography; SCr, serum creatinine.

Reference

1. Gao J, Yu QL, Fu RG, Wei LT, Wang M, Dong FM, Wang Z, Yang PT, Liu XH, Dai ZJ. Lack of Association Between Polymorphisms in AGT and ATR1 and IgA Nephropathy in a Chinese Population. Genet Test Mol Biomarkers. 2015; 19:710–13. https://doi.org/10.1089/gtmb.2015.0167

2. Moradi M, Rahimi Z, Amiri S, Rahimi Z, Vessal M, Nasri H. AT1R A1166C variants in patients with type 2 diabetes mellitus and diabetic nephropathy. J Nephropathol. 2015; 4:69–76. https://doi.org/10.12860/jnp.2015.14

3. Chen WJ, Huang YL, Shiue HS, Chen TW, Lin YF, Huang CY, Lin YC, Han BC, Hsueh YM. Renin-angiotensin-aldosterone system related gene polymorphisms and urinary total arsenic related to chronic kidney disease. Toxicol Appl Pharmacol. 2014; 279:95–102. https://doi.org/10.1016/j.taap.2014.05.011

4. Hanna MO, Shahin RM, Meshaal SS, Kostandi IF. Susceptibility and progression of end stage renal disease are not associated with angiotensin II type 1 receptor gene polymorphism. J Recept Signal Transduct Res. 2015; 35:381–85. https://doi.org/10.3109/10799893.2014.956757

5. Shah VN, Cheema BS, Sharma R, Khullar M, Kohli HS, Ahluwalia TS, Mohan V, Bhansali A. ACACβ gene (rs2268388) and AGTR1 gene (rs5186) polymorphism and the risk of nephropathy in Asian Indian patients with type 2 diabetes. Mol Cell Biochem. 2013; 372:191–98. https://doi.org/10.1007/s11010-012-1460-2

6. Möllsten A, Vionnet N, Forsblom C, Parkkonen M, Tarnow L, Hadjadj S, Marre M, Parving HH, Groop PH. A polymorphism in the angiotensin II type 1 receptor gene has different effec on the risk of diabetic nephropathy in men and women. Mol Genet Metab. 2011; 103:66–70. https://doi.org/10.1016/j.ymgme.2011.01.004

7. Zsom M, Fülöp T, Zsom L, Baráth A, Maróti Z, Endreffy E. Genetic polymorphisms and the risk of progressive renal failure in elderly Hungarian patients. Hemodial Int. 2011; 15:501–08 https://doi.org/10.1111/j.1542-4758.2011.00593.x

8. Huang HD, Lin FJ, Li XJ, Wang LR, Jiang GR. Genetic polymorphisms of the renin-angiotensin-aldosterone system in Chinese patients with end-stage renal disease secondary to IgA nephropathy. Chin Med J (Engl). 2010; 123:3238–42.

9. Kim SM, Chin HJ, Oh YK, Kim YS, Kim S, Lim CS. Blood pressure-related genes and the progression of IgA nephropathy. Nephron Clin Pract. 2009; 113:c301–08. https://doi.org/10.1159/000235948

10. Buraczynska M, Ksiazek P, Drop A, Zaluska W, Spasiewicz D, Ksiazek A. Genetic polymorphisms of the renin-angiotensin system in end-stage renal disease. Nephrol Dial Transplant. 2006; 21:979–83. https://doi.org/10.1093/ndt/gfk012

11. Prasad P, Tiwari AK, Kumar KM, Ammini AC, Gupta A, Gupta R, Sharma AK, Rao AR, Nagendra R, Chandra TS, Tiwari SC, Rastogi P, Gupta BL, Thelma BK. Chronic renal insufficiency among Asian Indians with type 2 diabetes: I. Role of RAAS gene polymorphisms. BMC Med Genet. 2006; 7:42. https://doi.org/10.1186/1471-2350-7-42

12. Fabris B, Bortoletto M, Candido R, Barbone F, Cattin MR, Calci M, Scanferla F, Tizzoni L, Giacca M, Carretta R. Genetic polymorphisms of the renin-angiotensin-aldosterone system ai renal insufficiency in essential hypertension. J Hypertens. 2005; 23:309–16. https://doi.org/10.1097/00004872-200502000-00013

13. Stratta P, Bermond F, Guarrera S, Canavese C, Carturan S, Dall'Omo A, Ciccone G, Bertola L, Mazzola G, Fasano E, Matullo G. Interaction between gene polymorphisms of nitric oxide synthase and renin-angiotensin system in the progression of membranous glomerulonephritis. Nephrol Dial Transplant. 2004; 19:587–95. https://doi.org/10.1093/ndt/gfg604

14. Woo KT, Lau YK, Choong LH, Zhao Y, Tan HB, Fook-Chong S, Tan EK, Yap HK, Wong KS. Polymorphism of renin-angiotensin system genes in IgA nephropathy. Nephrology

(Carlton). 2004; 9:304–09. https://doi.org/10.1111/j.1440-1797.2004.00291.x

15. Basset EA, Berthoux P, Cécillon S, Deprle C, Thibaudin D, De Filippis JP, Alamartin E, Berthou F. Hypertension after renal transplantation and polymorphism of genes involved in essential hypertension: ACE, AGT, AT1 R and ecNOS. Clin Nephrol. 2002; 57:192–200. https://doi.org/10.5414/CNP57192

16. Fradin S, Goulet-Salmon B, Chantepie M, Grandhomme F, Morello R, Jauzac P, Reznik Y. Relationship between polymorphisms in the renin-angiotensin system and nephropathy in typ[illegible] diabetic patients. Diabetes Metab. 2002; 28:27–32.

17. Losito A, Kalidas K, Santoni S, Ceccarelli L, Jeffery S. Polymorphism of renin-angiotensin system genes in dialysis patients—association with cerebrovascular disease. Nephrol Dial Transplant. 2002; 17:2184–88. https://doi.org/10.1093/ndt/17.12.2184

18. Thomas GN, Critchley JA, Tomlinson B, Lee ZS, Young RP, Cockran CS, Chan JC. Albuminuria and the renin-angiotensin system gene polymorphisms in type-2-diabetic and in normoglycemic hypertensive Chinese. Clin Nephrol. 2001; 55:7–15.

19. van Ittersum FJ, de Man AM, Thijssen S, de Knijff P, Slagboom E, Smulders Y, Tarnow L, Donker AJ, Bilo HJ, Stehouwer CD. Genetic polymorphisms of the renin-angiotensin system and complications of insulin-dependent diabetes mellitus. Nephrol Dial Transplant. 2000; 15:1000–07. https://doi.org/10.1093/ndt/15.7.1000

20. Wu S, Xiang K, Zheng T, Sun D, Weng Q, Zhao H, Li J. Relationship between the renin-angiotensin system genes and diabetic nephropathy in the Chinese. Chin Med J (Engl). 2000; 113:437–41.

21. Chowdhury TA, Dyer PH, Kumar S, Gough SC, Gibson SP, Rowe BR, Smith PR, Dronsfield MJ, Marshall SM, Mackin P, Dean JD, Morris PJ, Davies S, et al. Lack of association of angiotensin II type 1 receptor gene polymorphism with diabetic nephropathy in insulin-dependent diabetes mellitus. Diabet Med. 1997; 14:837–40. https://doi.org/10.1002/(SICI)1096-9136(199710)14:10<837::AID-DIA463>3.0.CO;2-V

22. Marre M, Jeunemaitre X, Gallois Y, Rodier M, Chatellier G, Sert C, Dusselier L, Kahal Z, Chaillous L, Halimi S, Muller A, Sackmann H, Bauduceau B, et al. Contribution of genetic polymorphism in the renin-angiotensin system to the development of renal complications in insulin-dependent diabetes: genetique de la Nephropathie Diabetique (GENEDIAB) study group. Clin Invest. 1997; 99:1585–95. https://doi.org/10.1172/JCI119321

23. Pei Y, Scholey J, Thai K, Suzuki M, Cattran D. Association of angiotensinogen gene T235 variant with progression of immunoglobin A nephropathy in Caucasian patients. J Clin Invest. 1997; 100:814–20. https://doi.org/10.1172/JCI119596
